# A phase I, placebo-controlled, randomized, double-blind, single ascending dose-ranging study to evaluate the safety and tolerability of a novel biophysical bronchodilator (S-1226) administered by nebulization in healthy volunteers

**DOI:** 10.1186/s13063-016-1489-8

**Published:** 2016-07-28

**Authors:** Francis H. Y. Green, Richard Leigh, Morenike Fadayomi, Gurkeet Lalli, Andrea Chiu, Grishma Shrestha, Sharif G. ElShahat, David Evan Nelson, Tamer Y. El Mays, Cora A. Pieron, John H. Dennis

**Affiliations:** 1 Department of Pathology & Laboratory Medicine, University of Calgary, Calgary, AB Canada; 2 Department of Medicine, University of Calgary, Calgary, AB Canada; 3 SolAeroMed Inc., Calgary, AB Canada

**Keywords:** Asthma, CO_2_, Perflubron, Phase I, Safety, S-1226, Biophysical drug

## Abstract

**Background:**

A major challenge in treating acute asthma exacerbations is the need to open constricted airways rapidly enough to reestablish ventilation and allow delivery of conventional medication to diseased airways. The solution requires a new approach that considers both biophysical and pharmacological aspects of treatments used in acute asthma. The result of testing several formulations was S-1226: carbon dioxide-enriched air delivered in nebulized perflubron, a synthetic surfactant. These agents act synergistically to rapidly reopen closed airways within seconds. The bronchodilator effect is independent of β-adrenergic and cholinergic mediated-signaling pathways, offering a unique mechanism of action. S-1226 has a low toxicity profile and was effective in treating bronchoconstriction in animal models of asthma. The goal of the present study was to evaluate the safety and tolerability of S-1226 in healthy human subjects.

**Methods:**

The phase I study was a single-center, randomized, double-blind, placebo-controlled, sequential, single-ascending-dose study conducted in Canada. Thirty-six subjects were distributed into three cohorts. Within each cohort, subjects were randomized to receive a single dose of S-1226 or a matching placebo administered over a 2-minute nebulization period. S-1226 was formulated with perflubron and 4 %, 8 %, or 12 % CO_2_. The dose of CO_2_ was sequentially escalated by cohort. The safety and tolerability of S-1226 were evaluated through assessment of adverse events, vital signs, 12-lead electrocardiograms, clinical laboratory parameters, and physical examinations.

**Results:**

S-1226 was safe and well tolerated at all three CO_2_ levels (4 %, 8 %, and 12 %). A total of 28 adverse events were reported, and all were judged mild in severity. Twenty-four adverse events occurred in the S-1226 cohort, of which five were considered remotely related and six possibly related to S-1226.

**Conclusions:**

S-1226 is a novel drug being developed for the treatment of acute asthma exacerbations. It consists of CO_2_-enriched air and perflubron and has potential to offer rapid and potent bronchodilation. The results of the study indicate that S-1226 is safe and well tolerated. All adverse events were mild, reversible, and likely due to known side effects of CO_2_ inhalation.

**Trial registration:**

ClinicalTrials.gov NCT02616770. Registered on 25 November 2015.

**Electronic supplementary material:**

The online version of this article (doi:10.1186/s13063-016-1489-8) contains supplementary material, which is available to authorized users.

## Background

According to the Global Asthma Report, the prevalence of asthma is 334 million individuals worldwide, and this number is projected to continue to rise [1]. Furthermore, an estimated 250,000 people die prematurely as a result of asthma each year. In most cases, these deaths are avoidable and could be prevented [2]. In 2011, 8.6 % of Canadians aged 12 years or older were reported to be diagnosed with asthma [3].

Healthcare costs of asthma are considered to be among the highest associated with chronic disease. In Canada, it is estimated that 65 % of patients with asthma treated in general practice have poorly controlled disease [4]. Furthermore, asthma is the leading cause of hospitalization, with the cost of urgent care (including hospitalizations, unscheduled physician and emergency department visits, medications, and ambulance services) associated with the disease being assessed at $46 million to $141 million per annum [3, 5]. Inhaled short-acting β_2_-agonists (with or without accompanying anticholinergic agents and systemic corticosteroids) represent the most common frontline emergency department treatment for acute exacerbations because of their well-known rapid bronchodilatory effect [6, 7]. However, many patients do not respond to these treatments, resulting in disproportionate consumption of healthcare resources [8].

In an acute asthma exacerbation, bronchoconstriction upon a background of mucous plugs, vascular congestion, inflammation, and airway remodeling characteristic of severe asthma can result in fatal hypoxemia. Autopsy examination reveals lungs characterized by areas of hyperinflation and collapse, as well as airways blocked by large, tenacious mucous plugs [9]. Poor response to initial therapy requires more aggressive strategies, with approximately one-third of patients with asthma admitted to intensive care units requiring intubation and mechanical ventilation [7, 10]. The latter treatment requires high pressure, resulting in barotrauma and associated complications [10].

The major challenge in treating acute asthma exacerbations is the need to open constricted airways rapidly enough to reestablish ventilation and allow delivery of oxygen and conventional medication to the diseased airways and to reduce the risk for barotrauma. It requires a new approach, one that takes into account both the biophysical and pharmacological aspects of acute asthma.

Our investigations resulted in the development of S-1226, an aerosol-vapor-gas mixture of CO_2_ and perflubron. CO_2_ and perflubron work together synergistically via both pharmacological and biophysical principles, offering a unique mechanism of action to rapidly dilate airways.

### Carbon dioxide

CO_2_ is an odorless, colorless, relatively inactive, nonflammable gas found in trace amounts in the atmosphere (0.038 %). It plays multiple roles in homeostasis in all living organisms and is essential for normal cellular function [11]. When CO_2_ is inhaled, or after it is produced by cellular respiration, it is dissolved into the blood, where it reacts with water. The carbonic enzyme that is formed when CO_2_ undergoes hydration in the bloodstream is used in renal function and in maintaining a proper internal pH balance. CO_2_ acts through the stimulation of chemoreceptors in the carotid bodies and respiratory control centers in the brain and brainstem [12] to increase breathing frequency and tidal volume [13]. CO_2_ also has a direct, dose-dependent, relaxant effect on vascular and bronchial smooth muscle [12, 14, 15], resulting in increased gas exchange and compliance within the lungs [16].

Inhaled CO_2_ is a known bronchodilator for many mammalian species [16]. In vitro studies have shown that hypercapnia relaxes airway smooth muscle [17, 18], whereas hypocapnia constricts it [19]. Similar effects are seen in vivo: Hypocapnia causes bronchoconstriction in healthy individuals and patients with asthma, whereas hypercapnia reduces airway resistance [20]. A study of young patients with atopic asthma demonstrated that inhalation of 6 % CO_2_ over 4–5 minutes relieved exercise-induced airflow obstruction, both at rest and during exercise, with no significant side effects reported [13].

### Perflubron

Perflubron is chemically an extremely stable, inert compound [21]. It is one of the most widely used perfluorocarbons and has been used extensively in clinical applications, including bronchial lavage, liquid ventilation, and gastrointestinal contrast-enhanced imaging, with no significant toxicity, as it is not metabolized and is minimally absorbed [21, 22]. It is hydrophobic and slightly less lipophobic than most perfluorocarbons of the same molecular weight, and it has among the greatest O_2_ and CO_2_ solubilities relative to its molecular weight [21, 23]. Perflubron also possesses high density and low surface tension, making it compatible with endogenous airway surfactant. It has mucolytic properties in vitro [24]. In pulmonary applications, perflubron is associated with improved lung compliance and gas exchange [25, 26]. In preclinical evaluations, aerosolized perflubron reduced pulmonary inflammation in addition to improving lung compliance and gas exchange [27, 28]. Thus, perflubron acts as the biophysical component of S-1226, and its effect is believed to be due to compatibility of perflubron with airway surfactant, its mucolytic properties, and its ability to absorb CO_2_. It may reduce inflammation and irritation by suppressing airway irritant receptors that cause bronchoconstriction.

### S-1226

S-1226, the combination of CO_2_-enriched ambient air delivered in nebulized perflubron, was shown to rapidly open obstructed airways in a sheep model of chronic allergic asthma with an effect that was greater and more prolonged than that of either CO_2_ or perflubron alone [29]. Moreover, studies using methacholine showed that S-1226 dilated constricted ovine airways within seconds, indicating a neural mechanism, potentially by activating noncholinergic, nonadrenergic nerves located between the epithelial cells in the bronchial mucosa [19].

The clinical indication for S-1226 is for the treatment of acute asthma exacerbations. It would complement existing rescue treatments for asthma, as it acts via a different mechanism to β_2_-agonists such as salbutamol or cholinergic antagonists [19]. Furthermore, the biophysical properties of S-1226 indicate that it will have mucolytic properties, thus providing relief when conventional therapies fail. If S-1226 is proven to be effective in humans, it may be possible to develop a portable rescue device similar to an EpiPen® (Mylan, Canonsburg, PA, USA) for patients with asthma at risk for an acute exacerbation. S-1226 also has potential for treating other obstructive airway diseases, such as chronic obstructive pulmonary disease and cystic fibrosis, and as a platform technology to enhance delivery of other drugs to the lung.

### Objective

In the present study, we sought to evaluate the safety and tolerability of S-1226, composed of 3 ml of perflubron with ascending doses of carbon dioxide (4 %, 8 %, and 12 % CO_2_) administered over a 2-minute nebulization period to healthy subjects. This phase I study is reported following the Consolidated Standards of Reporting Trials (CONSORT) 2010 checklist (Additional file 1).

## Methods

### Trial design

We conducted a single-center, phase I, randomized, double-blind, placebo-controlled, sequential, single-ascending-dose study in Canada. The study included three cohorts. Within each cohort, subjects were randomized to receive a single dose of S-1226 (nine subjects) or a matching placebo (three subjects). The dose of CO_2_ in S-1226 was sequentially escalated cohort by cohort from the starting dose of 4 % CO_2_. Planned subsequent dose levels were 8 % and 12 % CO_2_. Randomization within each cohort was imbalanced, with a study drug-to-placebo ratio of 3:1. The study was designed to evaluate the safety and tolerability of S-1226 in healthy male and female subjects under fasting conditions, with a total of 36 subjects equally distributed into three cohorts. Procedures completed during study screening, evaluation, and exit are detailed in Table 1.

**Table 1:** Study screening, evaluation, and exit procedures

Procedure	Screening	Study day 1 (check-in)	Study day 1 (confinement)	Study day 2 (checkout)	Study exit (follow-up visit day 5 ± 1)
Demographic data	X				
Medical and medication histories	X				
Review of AEs and concomitant medications		X			X
Physical examination	X	X		X	X
Height and weight	X				
Vital signs	X	X	X^a*^		X
Tympanic temperature	X	X			X
Spirometry^b^	X		X^c*^		
Chest x-ray	X				
ECG	X		X^d*^		X
Biochemistry	X	X	X^e*^	X	X
Hematology	X	X	X^f*^	X	X
HIV and hepatitis	X				
Urinalysis	X	X		X	X
Urine drug screen	X	X			
Urine cotinine test	X	X			
Alcohol breath test	X	X			
Serum pregnancy test	X	X			
Urine pregnancy test					X
Confinement		X	X		
Drug administration			X		
PK blood sample			X^e*^	X	
PK urine sample (for perflubron measurements)			X^g*^	X	
Pulse oximetry	X	X	X^a*^		
Adverse event monitoring		X	X	X	X

*AE* adverse event, *ECG* electrocardiogram, *PK* pharmacokinetic

* For study procedures scheduled at the same time point, the order of precedence is as follows:

i. Blood draws (hematology and biochemistry)

ii. ECG

iii. Vital signs and pulse oximetry

iv. Spirometry

^a^Blood pressure, heart rate, respiratory rate, and pulse oximetry: predose and 20 minutes, 1 h, 2 h, 3 h, and 4 h postdose

^b^For screening, spirometry was forced expiratory volume in 1 second (FEV_1_) and forced vital capacity; for confinement, spirometry was FEV_1_ only

^c^Spirometry within 1 h of drug administration (predose) and at 25 minutes, 1 h, 3 h, and 8 h (only if values before that are abnormal) after the end of study drug administration

^d^ECG predose and at 15 minutes, 1 h, 3 h, 4 h, and 8 h (only if values before that are abnormal) postdose

^e^Biochemistry and PK blood samples were collected predose and at 3 minutes, 30 minutes, 1 h, 2 h, 4 h, 8 h, 12 h, and 24 h postdose

^f^Hematology was done predose and at 30 minutes, 2 h, 8 h, and 24 h postdose

^g^Urine samples were collected over the following time intervals: predose and 0–4 h, 4–8 h, 8–12 h, and 12–24 h postdose

In each cohort, a sentinel group of two subjects was dosed on day 1: one sentinel was dosed with the test product (S-1226) and the other sentinel with the matching placebo. The remaining subjects of the same cohort were dosed at least 24 h after sentinel dosing with approval from the qualified investigator upon assessing the sentinel group. Following dosing of each cohort, safety and tolerability data were collected for at least 24 h postdose and evaluated by a safety monitoring committee. The subjects also returned for a follow-up visit 5 days (±1 day) after dosing. The safety monitoring committee, composed of Dr. Richard Leigh, Dr. Francis Green, and Dr. Xueyu (Eric) Chen, reviewed the results from each cohort before making a decision regarding continuation of the study at the next prescribed dose level, decreasing the next dose level, repeating a dose level, or whether to evaluate any additional dosage, based on consideration of the clinical significance of safety and tolerability parameters. There was at least a 7-day period between dosing at each dose level.

### Participants

The study population included nonsmoking male and female volunteers from 18 to 55 years of age with a body mass index between 18.5 and 30.0 kg/m^2^ who were judged to be healthy based on a medical history, electrocardiogram (ECG), laboratory evaluation, physical examination, vital sign measurements, pulse oximetry, spirometry, radiography, being capable of providing consent, and having normal lung function (forced expiratory volume in 1 second [FEV_1_] ≥ 80 % of predicted and FEV_1_, forced vital capacity >70 %).

Participants to whom any of the following applied were excluded from the study:

1. Any clinically significant abnormality or abnormal laboratory test results, including clinical ECG abnormalities, vital sign abnormalities, or positive test for hepatitis B, hepatitis C, or HIV found during medical or laboratory screening
2. History of significant allergic reactions, panic disorder or panic attacks, wheezing after exercise, and previous medical diagnosis of asthma
3. Positive urine drug screen or urine cotinine test at screening
4. Current cigarette smokers or former smokers with a smoking history >5 pack-years or who had stopped smoking within the 2 years preceding enrollment in the study
5. History of significant alcohol abuse within 6 months of the screening visit and/or a history of significant drug abuse within 1 year prior to screening
6. Use of anticoagulants, immunosuppressives, nonsteroidal anti-inflammatory drugs, anti-immunoglobulin E medication, or any allergen-specific immunotherapy within the last 60 days and use of medications other than nonsteroidal topical products without significant systemic absorption, such as prescription medication, over-the-counter products including natural health products, a depot injection or an implant of any drug, or monoamine oxidase inhibitors
7. Hemoglobin level <128 g/L (males) and <115 g/L (females) and hematocrit <0.37 L/L (males) and <0.32 L/L (females) at screening
8. Donation of blood within 7 days prior to dosing or blood loss ranging from 50 ml to >499 ml within 56 days prior to dosing
9. Breastfeeding subjects and participants with a positive pregnancy test at screening
10. Any reason that, in the opinion of the investigator (or delegate), would prevent the subject from participating in the study
11. Use of an investigational drug within 30 days (90 days for biologics) or participation in an investigational study within 30 days prior to dosing

### Locations where data were collected

The study took place at Pharma Medica Research Inc. (PMRI) in Toronto, ON, Canada. The clinical laboratory facility used was Alpha Laboratories Incorporated in Toronto.

### Interventions

S-1226 is formulated with 3 ml of perflubron and 4 %, 8 %, or 12 % CO_2_. Each formulation is inhaled directly from a nebulizer over a 2-minute administration period. Subjects received, under fasting conditions, a single dose of S-1226 or matching placebo administered over 2 minutes with a Circulaire® II hybrid nebulizer system (Westmed, Tucson, AZ, USA) at a flow rate of 8.9 L/minute. The nebulizer was filled with either 3 ml of perflubron driven by a compressed medical gas mixture containing CO_2_ (S-1226) or 3 ml of normal saline driven with compressed medical air (placebo). The design of the Circulaire® nebulizer system incorporates an inflatable reservoir bag, which collects nebulized gas during the exhalation phase so that during the inhalation phase, and the subject receives both freshly generated nebulized drug and stored nebulized drug at a sufficient rate. Thus, no ambient air is entrained into the system, ensuring that nebulized gas concentrations are maintained throughout the breathing cycle.

Three S-1226 formulations were tested:

1. S-1226 (4 %): composed of perflubron and 4 % CO_2_
2. S-1226 (8 %): composed of perflubron and 8 % CO_2_
3. S-1226 (12 %): composed of perflubron and 12 % CO_2_

Each S-1226 formulation was administered by inhalation for a period of 2 minutes, with 0 h defined as the end of nebulization. Subjects were allowed to stop treatment at any time at the appearance of adverse events (AEs), including intolerable anxiety or panic.

Subjects were confined in the clinical site for 24 h following administration of S-1226 for procedural and safety measures. Normally, CO_2_ is eliminated from the body via exhalation at the same rate at which it is produced. In addition, perflubron is an extremely stable and inert compound that is excreted as a vapor during exhalation. Therefore, a 24-h confinement was long enough to collect and monitor any AEs that might occur.

No food was allowed from at least 10 h before dosing until at least 2 h after dosing. Controlled meals were served at appropriate times during the remainder of the confinement period. Meals were similar in composition for all cohorts. The evening before dosing, subjects were asked to drink at least two glasses of water (approximately 240 ml each). On the morning of dosing, subjects were asked to drink at least one glass of water (approximately 240 ml) at least 1 h predose. Starting at 1 h predose, no fluids were allowed until 1 h postdose. At 1 h after study treatment, subjects were asked to drink at least one glass (approximately 240 ml) of water every 4 h over the 12-h period following study treatment. Thereafter, water was provided ad libitum.

Safety parameters, including laboratory results and ECG, were assessed by the qualified investigator or delegate. In making the medical assessment, the clinical site criteria for laboratory and ECG acceptance ranges were used as suggested guidelines. Scheduled safety measurements were repeated according to the clinical site standard operating procedures or upon request from a physician. Any abnormal measurement was evaluated by a physician and repeated if judged necessary. No pharmacokinetic assessments of perflubron were performed, as the dose of perflubron in S-1226 is below the detection limit of headspace gas chromatography with electron capture detection, the most sensitive analytical technique to measure perflubron in the blood and urine.

### Primary and secondary outcome measures

Safety and tolerability to S-1226 were evaluated through the assessment of AEs, vital signs (respiratory rate, blood pressure, and heart rate), 12-lead ECG, clinical laboratory parameters (spirometry), and physical examination, as detailed in Table 1. Safety and tolerability data were reported using descriptive statistics.

The data captured for this study allowed for adequate evaluation of the safety of the subjects after product administration. These data (AEs, vital signs, 12-lead ECG, clinical laboratory parameters, and physical examination) were reviewed by the qualified investigator throughout the study and by the safety monitoring committee after the completion of cohorts 1 and 2. On the basis of the results of the documented measurements, the safety monitoring committee determined that all subsequent dosing could proceed according to the protocol. The collected measurements provided the qualified investigator and safety monitoring committee adequate data to assess the subjects objectively and provide a study conclusion. The efficacy of the tested products was not evaluated for this study.

### Sample size

The sample size of 12 subjects per cohort is a number commonly used in first-in-human studies, and it was judged appropriate to achieve the study’s objectives. A total of 36 subjects were recruited into the study.

### Randomization and blinding

The randomization scheme was generated by PMRI. Participants were randomly assigned using the randomization procedures to either the S-1226 or the placebo group within each cohort. Randomization was carried out by using a computer-generated random list of numbers prepared and checked by designated personnel with no direct involvement with the clinical aspects of the trial. The numbered list was generated separately for each cohort before administration of the drug, and labels were placed on the unit dose package.

Both study subjects and the clinical personnel involved in the collection, monitoring, revision, or evaluation of AEs, as well as personnel who could have an impact on the outcome of the study, were blinded with respect to subject treatment assignment until the clinical phase of the study was completed (i.e., when investigation and reporting of all AEs were completed). The study personnel responsible for subject dosing were not involved in the collection, monitoring, revision, or evaluation of AEs. Designated personnel at the clinical site not directly involved with the clinical aspects of the trial prepared (including loading the nebulizer) and dispensed the study medication and were aware of the randomization code. Before administration of the drug to the participant, the appropriate individual subject randomization envelope was opened, which revealed which treatment was to be administered. Research pharmacists dispensed either the drug or placebo according to the randomization list.

In the event of an emergency, an envelope for each subject containing the subject’s treatment assignment was available from the clinical site personnel involved with the preparation of the study medication. The qualified investigator or other attending study physician (subinvestigator) was required to make every effort to contact the sponsor prior to unblinding a subject’s treatment assignment and to record the date and reason for the unblinding in the study source documents.

To avoid compromising blinding of the study, S-1226 and placebo had the same visual appearance, and the same model of hospital nebulizer was used for administration of both compounds. The compressed gas mixtures were similarly blinded. The perflubron and placebo (saline) used in the study were delivered to the clinical trial facility in different forms. The perflubron was contained in 3-ml sterile bottles, while the saline was delivered in ampoules. Apart from the packaging, both were in liquid form and had the same visual appearance. Before administration to the participants, both S-1226 and placebo were repackaged into 51.75 ml snap-cap amber vials and consecutively numbered for each participant according to the randomization schedule. Each participant was assigned an order number and received the drugs in the corresponding prepacked bottle. The same model of hospital nebulizer was used for administration of both compounds.

### Ethical conduct of the study

Trained personnel obtained written informed consent from each participant prior to enrolling the participant in the study. Participants were permitted to withdraw from the study at any time. This phase I study received ethical approval from the Optimum Clinical Research Inc. Institutional Review Board. This study was conducted in accordance with the following:

1. Current Health Canada Therapeutic Products Directorate guidance documents
2. Good Clinical Practice as established by the International Conference on Harmonization
3. Basic principles defined in the U.S. Code of Federal Regulations (21 CFR Part 312)
4. Principles enunciated in the World Medical Association Declaration of Helsinki (October 2013)

### Informed consent

All subjects signed a privacy consent form prior to disclosing any personal information and prior to undergoing any medical procedures. Upon study entry, subjects were given a copy of the current informed consent form to read and were given the opportunity to ask questions. When all questions were satisfactorily answered, subjects were asked to sign the informed consent form prior to study initiation.

### Statistical methods

The primary endpoint of the study was to evaluate the safety and tolerability of S-1226, composed of perflubron with ascending doses of CO_2_ (4 %, 8 %, and 12 % CO_2_), administered to healthy subjects over a 2-minute nebulization period. The study had no secondary efficacy endpoints.

Descriptive statistics were presented for demographic parameters by treatment group and for all other parameters. Treatment-emergent AEs were summarized descriptively by treatment for all subjects who were dosed (safety population). No inferential statistical analysis of safety data was planned. Safety and tolerability data were reported using descriptive statistics.

## Results

**Fig. 1:**
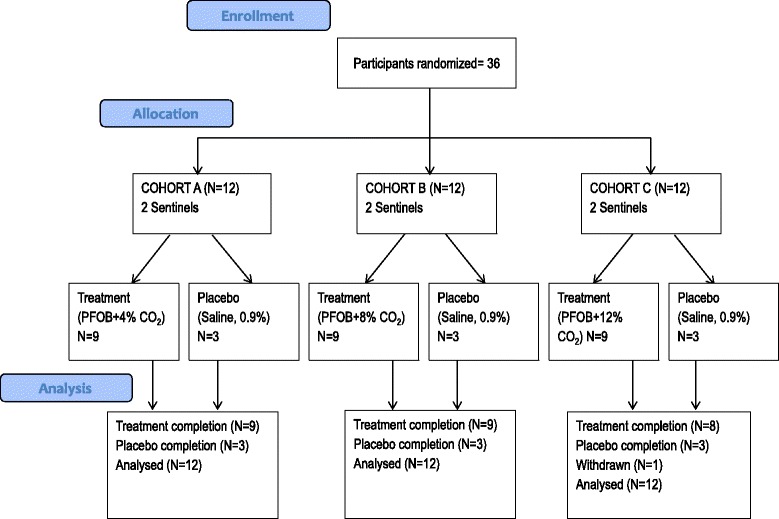
2010 Consolidated Standards of Reporting Trials (CONSORT) flow diagram. Participant enrollment, allocation, and analysis in S-1226 (4 %, 8 %, and 12 % CO_2_) and placebo treatment groups. *PFOB* perflubron

Participants were recruited in March 2014, and the study was completed on 17 May 2014. The final report of the study was completed in September 2014. The study had three cohorts, with each cohort receiving a different concentration of CO_2_ in the S-1226 treatment. Overall, nine participants were randomly assigned to receive the placebo (saline), nine to receive S-1226 (4 % CO_2_), nine to receive S-1226 (8 % CO_2_), and nine to receive S-1226 (12 % CO_2_), as summarized in Fig. 1. The treatment groups were comparable for most demographic and baseline characteristics (Table 2).

**Table 2:** Baseline patient demographic and clinical characteristics

	Cohort 1		Cohort 2		Cohort 3	
	S-1226 (4 % CO_2_) (*n* = 9)	Placebo (*n* = 3)	S-1226 (8 % CO_2_) (*n* = 9)	Placebo (*n* = 3)	S-1226 (12 % CO_2_) (*n* = 9)	Placebo (*n* = 3)
Age, years						
Mean ± SD	35 ± 12	35 ± 11	35 ± 10	31 ± 3	33 ± 10	27 ± 11
Median	30	36	32	32	30	23
Range	22–55	23–45	22–51	28–34	21–45	19–39
Height, cm						
Mean ± SD	170.2 ± 7.1	166.7 ± 4.2	168.0 ± 10.7	171.3 ± 2.8	166.4 ± 8.8	186.0 ± 6.5
Median	170.7	164.6	164.6	170.5	165.5	183.9
Range	155.7–182.2	164.0–171.5	155.4–184.5	169.0–174.5	153.0–181.4	180.8–193.2
Weight, kg						
Mean ± SD	73.5 ± 12.4	69.4 ± 13.7	69.3 ± 13.2	67.4 ± 9.5	65.3 ± 9.1	81.3 ± 12.7
Median	75.3	63.4	65.0	62.2	62.0	75.5
Range	58.4–96.8	59.7–85.1	53.1–94.0	61.6–78.4	51.9–78.0	72.6–95.9
BMI, kg/m^2^						
Mean ± SD	25.3 ± 3.6	24.8 ± 3.6	24.4 ± 2.5	22.9 ± 2.4	23.6 ± 2.5	23.6 ± 4.3
Median	26.5	23.4	24.9	21.8	23.7	22.2
Range	20.0–29.2	22.2–28.9	19.5–28.5	21.2–25.7	19.2–26.4	20.2–28.4
Age group, years, *n* (%)						
< 18	0 (0 %)	0 (0 %)	0 (0 %)	0 (0 %)	0 (0 %)	0 (0 %)
18–40	7 (77.8 %)	2 (66.7 %)	7 (77.8 %)	3 (100 %)	6 (66.7 %)	3 (100 %)
41–55	2 (22.2 %)	1 (33.3 %)	2 (22.2 %)	0 (0 %)	3 (33.3 %)	0 (0 %)
Sex, *n* (%)						
Female	2 (22.2 %)	3 (100 %)	5 (55.6 %)	2 (66.7 %)	8 (88.9 %)	0 (0 %)
Male	7 (77.8 %)	0 (0 %)	4 (44.4 %)	1 (33.3 %)	1 (11.1 %)	3 (100 %)
Ethnicity, *n* (%)						
Hispanic or Latino	2 (22.2 %)	1 (33.3 %)	3 (33.3 %)	2 (66.7 %)	4 (44.4 %)	2 (66.7 %)
Not Hispanic or Latino	7 (77.8 %)	2 (66.7 %)	6 (66.7 %)	1 (33.3 %)	5 (55.6 %)	1 (33.3 %)
Race, *n* (%)						
Black	5 (55.6 %)	1 (33.3 %)	4 (44.4 %)	0 (0 %)	3 (33.3 %)	0 (0 %)
Other (multiracial white and black)	0 (0 %)	1 (33.3 %)	0 (0 %)	0 (0 %)	0 (0 %)	0 (0 %)
White	4 (44.4 %)	1 (33.3 %)	5 (55.6 %)	3 (100 %)	6 (66.7 %)	3 (100 %)
Heart rate, beats/minute, mean ± SD	66.2 ± 10.2	71.7 ± 7.5	61.9 ± 5.4	72.7 ± 9.5	72.3 ± 12.0	70.7 ± 7.4
FEV_1_, L/minute, mean ± SD	3.4 ± 0.7	2.8 ± 0.2	3.5 ± 1.0	3.9 ± 0.4	3.3 ± 0.7	4.9 ± 0.9
Blood pressure, mmHg,^a^ mean ± SD	87.3 ± 8.0	89.7 ± 5.7	85.6 ± 8.0	89.2 ± 9.4	82.8 ± 9.0	87.0 ± 2.4

*BMI* body mass index, *FEV*_*1*_ forced expiratory volume in 1 second

^a^Blood pressure values in participants were expressed as mean arterial pressures

There was a minor protocol modification in the study. Spirometry training was scheduled to be performed on the night of check-in, and the predose spirometry measurement was scheduled to be performed within 1 h prior to drug administration. After discussions with the sponsor and the investigator, owing to tight time constraints, the predose spirometry test was performed within 2 h prior to drug administration for cohort 2 and cohort 3. This modification had no impact on the safety of the subjects.

One participant in cohort 3 was discontinued from the study and was not replaced. The participant had shortness of breath during dosing and was unable to maintain a tight mouth seal. This participant’s shortness of breath was deemed to have a possible relationship to the drug. However, this participant was included in safety data analyses.

### Baseline characteristics

Baseline patient demographic and clinical characteristics are summarized in Table 2.

### Safety

The safety monitoring committee reviewed all relevant safety data upon completion of the first cohort. All committee members unanimously agreed to proceed to the next dosing levels of 8 % and 12 % without concern. A total of 28 AEs were reported throughout the study. All AEs were adjudged mild in severity. Thirteen subjects (48.1 %) reported twenty-four AEs after receiving the test product, and three (33.3 %) subjects reported four AEs after receiving the placebo product (Table 3).

**Table 3:** Adverse events by severity, relationship to drug, and action taken

	S-1226 (4 % CO_2_) (*n* = 9)	S-1226 (8 % CO_2_) (*n* = 9)	S-1226 (12 % CO_2_) (*n* = 9)	Placebo (*n* = 9)	Total (*n* = 36)
Severity					
Mild	3	1	20	4	28
Moderate	0	0	0	0	0
Severe	0	0	0	0	0
Relationship to drug					
Probable	0	0	0	0	0
Possible	1	0	6	0	7
Remote	1	1	3	1	6
Unrelated	1	0	11	3	15
Action taken					
Dose increased	0	0	0	0	0
Dose not changed	3	1	18	2	24
Dose reduced	0	0	0	0	0
Drug interrupted	0	0	1	0	1
Drug withdrawn	0	0	0	0	0
Not applicable	0	0	1	0	3
Unknown	0	0	0	0	0

Three AEs (10.7 %) were reported with an S-1226 dose of 4 % CO_2_, one AE (5.0 %) with 8 % CO_2_, and twenty AEs (71.4 %) with 12 % CO_2_. AEs were observed in system organ classes of cardiac disorders (*n* = 4); general disorders and administration site conditions (*n* = 3); investigations (*n* = 6); nervous system disorders (*n* = 8); respiratory, thoracic, and mediastinal disorders (*n* = 6); and vascular disorders (*n* = 1). The most frequent AE was somnolence, which was reported by four subjects (two at 4 % CO_2_, one at 8 % CO_2_, and one at 12 % CO_2_) (Table 4). Other commonly reported AEs were dyspnea, dizziness, bradycardia, and feeling hot.

**Table 4:** Incidence of adverse events by system organ class and preferred terms

System organ class	Preferred term	S-1226 (4 % CO_2_) (*n* = 9)	S-1226 (8 % CO_2_) (*n* = 9)	S-1226 (12 % CO_2_) (*n* = 9)	Placebo (*n* = 9)	Total (*n* = 36)
Subjects with one or more adverse events		3 (33.3 %)	1 (11.1 %)	9 (100 %)	3 (33.3 %)	16 (44.4 %)
Subjects with no adverse events		6 (66.7 %)	8 (88.9 %)	0 (0 %)	6 (66.7 %)	20 (55.6 %)
Cardiac disorders	Bradycardia	1 (11.1 %)	0 (0 %)	1 (11.1 %)	0 (0 %)	2 (5.6 %)
	Palpitations	0 (0 %)	0 (0 %)	0 (0 %)	1 (11.1 %)	1 (2.8 %)
	Hypertension	0 (0 %)	0 (0 %)	1 (11.1 %)	0 (0 %)	1 (2.8 %)
	Subtotal	1 (11.1 %)	0 (0 %)	2 (22.2 %)	1 (11.1 %)	4 (11.1 %)
General disorders and administration site conditions	Feeling hot	0 (0 %)	0 (0 %)	3 (33.3 %)	0 (0 %)	3 (8.3 %)
	Subtotal	0 (0 %)	0 (0 %)	3 (33.3 %)	0 (0 %)	3 (8.3 %)
Investigations	Neutrophil count decreased	0 (0 %)	0 (0 %)	2 (22.2 %)	1 (11.1 %)	3 (8.3 %)
	White blood cell count decreased	0 (0 %)	0 (0 %)	0 (0 %)	1 (11.1 %)	1 (2.8 %)
	Electrocardiogram QT prolongation	0 (0 %)	0 (0 %)	1 (11.1 %)	0 (0 %)	1 (2.8 %)
	Electrocardiogram PR prolongation	0 (0 %)	0 (0 %)	0 (0 %)	1 (11.1 %)	1 (2.8 %)
	Subtotal	0 (0 %)	0 (0 %)	3 (33.3 %)	3 (33.3 %)	6 (16.7 %)
Nervous system disorders	Dizziness	0 (0 %)	0 (0 %)	3 (33.3 %)	0 (0 %)	3 (8.3 %)
	Headache	0 (0 %)	0 (0 %)	1 (11.1 %)	0 (0 %)	1 (2.8 %)
	Somnolence	2 (22.2 %)	1 (11.1 %)	1 (11.1 %)	0 (0 %)	4 (11.1 %)
	Subtotal	2 (22.2 %)	1 (11.1 %)	5 (55.6 %)	0 (0 %)	8 (22.2 %)
Respiratory, thoracic, and mediastinal disorders	Cough	0 (0 %)	0 (0 %)	1 (11.1 %)	0 (0 %)	1 (2.8 %)
	Dyspnea	0 (0 %)	0 (0 %)	3 (33.3 %)	0 (0 %)	3 (8.3 %)
	Respiratory tract irritation	0 (0 %)	0 (0 %)	1 (11.1 %)	0 (0 %)	1 (2.8 %)
	Throat irritation	0 (0 %)	0 (0 %)	1 (11.1 %)	0 (0 %)	1 (2.8 %)
	Subtotal	0 (0 %)	0 (0 %)	6 (66.7 %)	0 (0 %)	6 (16.7 %)
Vascular disorders	Tachycardia	0 (0 %)	0 (0 %)	1 (11.1 %)	0 (0 %)	1 (2.8 %)
	Subtotal	0 (0 %)	0 (0 %)	1 (11.1 %)	0 (0 %)	1 (2.8 %)

### Treatment and placebo comparison

**Fig. 2:**
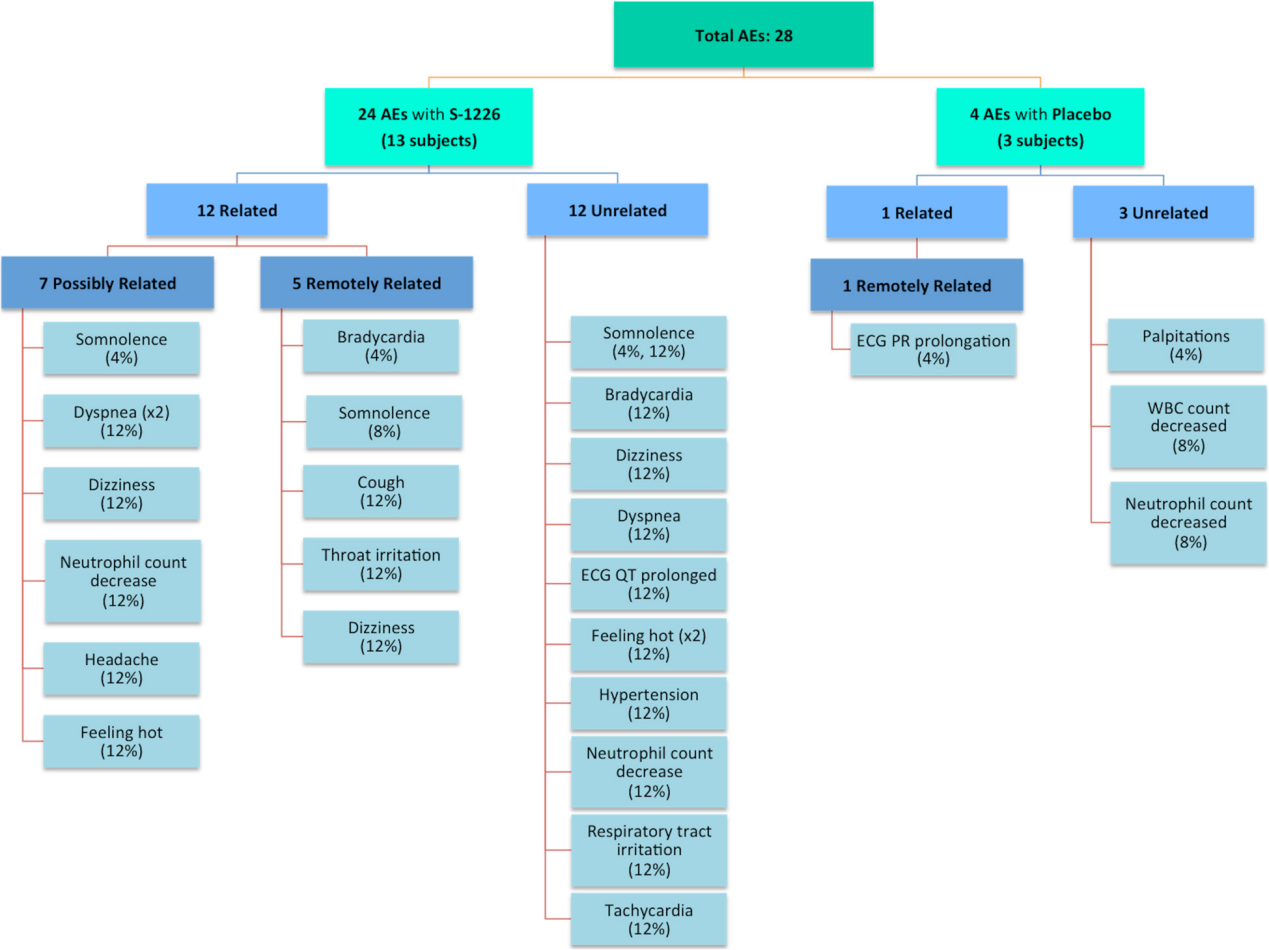
Flow diagram of related adverse events (AEs) in S-1226 (4 %, 8 %, and 12 % CO_2_) and placebo treatment groups based on their relationship to the drug (possibly or remotely). *ECG* electrocardiogram, *WBC* white blood cell

The number of AEs reported after administration of S-1226 (4 % CO_2_) (*n* = 3), S-1226 (8 % CO_2_) (*n* = 1), and placebo (*n* = 4) appeared to be similar, while subjects who received S-1226 (12 % CO_2_) reported a total of 20 AEs (Table 3). All nine subjects (100 %) who received S-1226 (12 % CO_2_) reported at least one AE. Of the 20 (71.4 %) reported AEs, 6 were considered possibly related to the drug, while 3 AEs were remotely related to S-1226 (Table 3, Fig. 2). All 20 AEs were adjudged mild in severity and were spread across several system organ classes and preferred term groups (Table 4). Therefore, there was not one AE that was prominently experienced in this study. Clinical review of the laboratory, ECG, vital sign, and physical examination data did not indicate any safety concerns of the investigator or safety monitoring committee.

## Discussion

The goal of this study was to evaluate the safety and tolerability of S-1226 at ascending doses of carbon dioxide (4 %, 8 %, and 12 % CO_2_) in healthy subjects. Twenty-eight AEs were observed in total, with the majority occurring at the highest dose (12 % CO_2_) of S-1226. The number of AEs reported after administration of S-1226 (4 % CO_2_), S-1226 (8 % CO_2_), and placebo appeared to be similar, showing that S-1226 was safe and well tolerated at these concentrations. At the highest dose of S-1226 (12 % CO_2_), 20 AEs were reported, suggesting that the risk of an AE increases as the dose of CO_2_ is increased. All AEs were mild in severity and were reversible within minutes to hours of the treatment. S-1226, a formulation of CO_2_-enriched ambient air delivered in nebulized perflubron, appeared safe and well tolerated at all three CO_2_ levels (4 %, 8 %, and 12 %) when administered to healthy volunteers.

In total, of the 24 AEs that occurred with the administration of S-1226, 5 were considered remotely related and 7 were considered possibly related to S-1226, as adjudged by the safety monitoring committee. These AEs are likely attributable to the known effects of CO_2_ inhalation and not perflubron, as the amount of perflubron was the same for each cohort while the number of AEs increased with increasing dose of CO_2_. Moreover, most of these AEs, including somnolence, bradycardia, dizziness, feeling hot, headache, cough, and dyspnea are well-known side effects of CO_2_ inhalation, and the participants in the study were made aware of these risks while their informed consent was being obtained [15, 30]. In published clinical studies of CO_2_ administered at concentrations ranging from 2 % to 14 % over 2.5 minutes to 90 minutes, observed side effects, including neurological symptoms, were mostly mild but could become severe with prolonged administration of over 10 minutes of approximately 8 % CO_2_ or more. Overall, AEs become apparent with inhalation of >5 % CO_2_ for 4–35 minutes [14, 31, 32]. Symptoms most commonly reported were dyspnea, headaches, sweating, and dizziness. Alterations in mental state (e.g., restlessness, confusion) and feeling faint occurred more commonly at doses ≥10 % CO_2_. Loss of consciousness occurred with longer administration times (>8 minutes where reported) and at CO_2_ doses >10 % CO_2_ [14, 15, 33]. These effects are dose-dependent and reversible, returning to normal soon after treatment cessation. CO_2_ levels are highly regulated, leading to a relatively rapid resolution of effects observed after inhalation of concentrations greater than normally occur in arterial blood and alveoli. In this study, CO_2_ concentrations of 4 %, 8 %, and 12 % were delivered for 2 minutes to reduce the risk of symptoms associated with higher concentrations and extended delivery of CO_2_.

Furthermore, inhaled 6 % CO_2_ has been shown to relieve airway obstruction with no reported AEs in patients with asthma who experience exercise-induced bronchospasm [13]. On the basis of this finding and prior clinical research with inhaled CO_2_ and perflubron, including the presence at least 5 % CO_2_ dissolved in perflubron within the lungs of patients treated with perflubron as bronchial lavage or a partial liquid ventilation agent, S-1226 (4 % CO_2_) administered for 2 minutes was selected as a safe starting dose. Following a safety evaluation of this concentration, 8 % CO_2_ and 12 % CO_2_ were subsequently delivered to subjects, with previous preclinical animal studies supporting that they would be safe and effective to administer for 2 minutes. This range of doses of CO_2_ was chosen on the basis of safety studies in humans indicating that 12 % is the upper limit and efficacy studies in animal models of asthma.

On the basis of previous clinical research, the AEs in this study are likely accounted for as known side effects of CO_2_ inhalation and not related to perflubron. The toxicology profile of perflubron is well researched, and it is considered safe to use. Perflubron is biologically inert and is not metabolized [21]. It is a synthetic surfactant that has been administered to humans in high doses orally, intravenously, and by liquid instillation directly into the lungs. The majority of perflubron instilled into the lungs is eliminated from the body through respiration, as perflubron readily evaporates from surface tissues and is exhaled. In addition, very small proportions of the perflubron instilled in the respiratory tract partitions into blood [34]. It has been shown to improve lung compliance and gas exchange in pulmonary applications, and it has been marketed as a gastrointestinal imaging agent in the United States (Imagent® GI; Alliance Pharmaceutical Corp., San Diego, CA, USA) using a dose of 9 ml of perflubron per kilogram of body weight and as a bronchial lavage solution in Canada (Liquivent®, previously called Perflubronc®-B; OriGen Biomedical, Austin, TX, USA), showing it is safe to use and well tolerated by humans [35].

Both components of S-1226, perflubron and CO_2_, have undergone extensive clinical testing as separate agents, and their safety profiles are well known. CO_2_ and perflubron do not chemically interact. Due to the natural elimination of CO_2_ by respiration, clinical liquid ventilation studies with large volumes of perflubron, as well as clinical use of perflubron as a licensed bronchial lavage solution (Liquivent®), have involved levels of approximately 5 % or greater CO_2_ dissolved in perflubron within the lungs.

S-1226 is intended for clinical use as a single-dose treatment for acute asthma exacerbations. If multiple treatments are indicated in future studies, additional safety studies will be required. S-1226 is to be used within hospital emergency rooms, where rapid treatment administration and immediate relief of acute airway obstruction are vital. S-1226 has a unique mechanism of action independent of the β-adrenergic and cholinergic mechanism of conventional therapies and offers a novel treatment in cases where these other therapies have proven ineffective. Moreover, S-1226 dilates airways within seconds and can be combined with traditional medications and oxygen therapy to provide more effective and sustained relief following an exacerbation. In the future, if S-1226 is shown to be effective in humans, it may be possible to develop a rescue device similar to an EpiPen® (Mylan) for patients with asthma to be used following an acute asthma exacerbation to rapidly dilate the airways. S-1226 may also serve as a platform technology to enhance delivery of other drugs to the lung.

On the basis of this phase I trial demonstrating the safety and tolerability of S-1226 in humans, as well as preclinical studies that have shown the efficacy of S-1226 in rapidly dilating the airways in sheep and rat models of asthma, a phase II trial designed to determine the efficacy of S-1226 in patients with mild atopic asthma has been developed and is currently ongoing. In this single-dose, placebo-controlled crossover trial, a 8 % CO_2_ dose will be used. This dose was chosen because it produced the fewest AEs in the phase I trial.

## Conclusions

This phase I study shows that S-1226 is safe and well tolerated when administered to healthy humans at 4 %, 8 %, and 12 % CO_2_ nebulized in 3 ml of perflubron. All AEs associated with S-1226 were mild and reversible and were likely due to the known side effects of CO_2_ inhalation. S-1226 has the potential to be a rescue therapy for acute asthma exacerbations.

## Abbreviations

AE, adverse event; BMI, body mass index; ECG, electrocardiogram; FEV_1_, forced expiratory volume in 1 second; PFOB, perflubron; PK, pharmacokinetic; PMRI, Pharma Medica Research Inc.; WBC, white blood cell

## Additional file

Additional file 1: CONSORT 2010 checklist. (DOC 216 kb)
